# Spectroscopic Techniques for Monitoring Thermal Treatments in Fish and Other Seafood: A Review of Recent Developments and Applications

**DOI:** 10.3390/foods9060767

**Published:** 2020-06-10

**Authors:** Abdo Hassoun, Karsten Heia, Stein-Kato Lindberg, Heidi Nilsen

**Affiliations:** Nofima AS Norwegian Institute of Food, Fisheries, and Aquaculture Research Muninbakken 9-13, 9291 Tromsø, Norway; karsten.heia@nofima.no (K.H.); stein-kato.lindberg@nofima.no (S.-K.L.); heidi.nilsen@nofima.no (H.N.)

**Keywords:** control, cooking, fish, fluorescence, heat, hyperspectral, quality

## Abstract

Cooking is an important processing method, that has been used since ancient times in order to both ensure microbiological safety and give desired organoleptic properties to the cooked food. Fish and other seafood products are highly sensitive to thermal treatments and the application of severe heat can result in negative consequences on sensory and nutritional parameters, as well as other quality attributes of the thermally processed products. To avoid such undesired effects and to extend the shelf life of these perishable products, both the heat processing methods and the assessment techniques used to monitor the process should be optimized. In this review paper, the most common cooking methods and some innovative ones will first be presented with a brief discussion of their impact on seafood quality. The main methods used for monitoring heat treatments will then be reviewed with a special focus on spectroscopic techniques, which are known to be rapid and non-destructive methods compared to traditional approaches. Finally, viewpoints of the current challenges will be discussed and possible directions for future applications and research will be suggested. The literature presented in this review clearly demonstrates the potential of spectroscopic techniques, coupled with chemometric tools, for online monitoring of heat-induced changes resulting from the application of thermal treatments of seafood. The use of fluorescence hyperspectral imaging is especially promising, as the technique combines the merits of both fluorescence spectroscopy (high sensitivity and selectivity) and hyperspectral imaging (spatial dimension). With further research and investigation, the few current limitations of monitoring thermal treatments by spectroscopy can be addressed, thus enabling the use of spectroscopic techniques as a routine tool in the seafood industry.

## 1. Introduction

Due to the high perishability of fish and other seafood products, a wide range of processing and conservation methods have been employed to ensure safety and extend the shelf life of these products [1,2,3]. Thermal treatments have been extensively used for a long time in order to improve palatability and give treated products desired organoleptic properties, along with eliminating or deactivating foodborne pathogens or spoilage bacteria. In recent years, due to the increase in demand for processed ready-to-eat food products, the thermal treatments have become even more important for processing aquatic products and other food. Nowadays, most processed food should be subjected to some kind of thermal treatment during processing or preparation prior to consumption [4,5]. However, application of severe heat treatments could result in quality deterioration, especially for foods with high contents of protein, such as fish and other seafood, which are known for their high sensitivity to high thermal load [6,7]. The effects of different thermal treatments on color, texture, water holding capacity and other quality parameters have been well-documented [8,9]. Additionally, inadequate cooking method, time, temperature, or time/temperature combinations could cause protein denaturation and aggregation, and protein and lipid oxidation, leading to, among others, a decreased protein digestibility [10,11]. Therefore, from a technological perspective, optimization of heat treatment serves as a key element in saving energy and retaining the maximum nutritional quality of the heat-treated products. Hence, in recent years, minimal processing [12], and more particularly minimal heat processing [13] which extends the shelf life of seafood while maintaining high levels of its quality and safety, has gained momentum.

**Figure 1:**
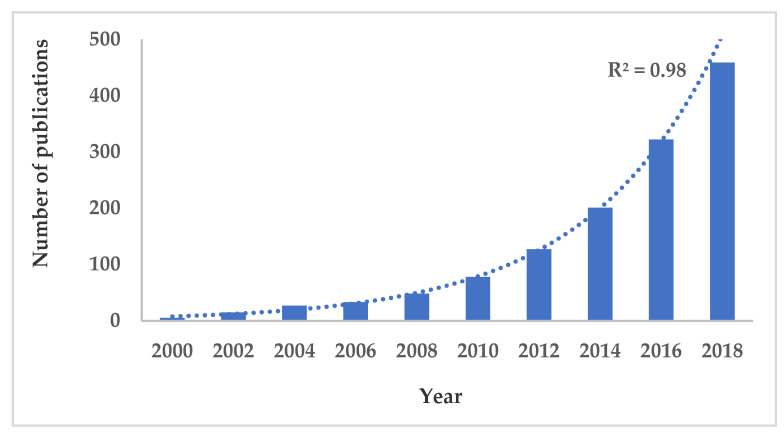
Cumulative number of studies investigating the impact of thermal treatments on fish and meat using spectroscopic techniques since the year 2000. Information obtained from the database Scopus (Search criteria: All fields: Thermal treatments, AND Article title, Abstract, Keywords: Spectroscopy, AND Article title, Abstract, Keywords: “Fish AND/OR Meat). The data were obtained on 22 May 2020.

In addition to sensory and microbiological analysis, several analytical techniques and instruments (e.g., texture profile analyzer for measuring the texture, colorimeter for evaluating the color, physical and chemical properties, etc.) have been applied for assessing heat-induced changes resulting from the application of thermal treatments. However, most of these methods and techniques are subjected to certain limitations, as they are time-consuming and destructive, thus cannot be applied online/inline during the industrial cooking process. Therefore, the devolvement of rapid, noncontact, non-destructive and real-time measurements, such as spectroscopic analysis, is of utmost importance for the modern food industry. According to the Scopus database, the number of publications regarding use of spectroscopic methods for monitoring thermal treatments in muscle food products is trending upwards (Figure 1). This number has increased from only 9 publications in the year 2000 to more than 160 papers in the year 2018. Two reasons can explain this exponential trend. Firstly, the growing awareness in the food industry and research of the importance of these techniques compared to traditional methods. In fact, unlike conventional methods, spectroscopic techniques represent promising tools for conducting rapid and non-destructive measurements, and hence can be more easily implemented into processing and production lines. Moreover, the spectroscopic techniques, as non-targeted methods, enable a detailed profile and complex chemical information to be obtained, meaning that the collected spectrum can be considered as a fingerprint of the examined sample [14,15,16]. Secondly, the commercial availability of spectroscopic instruments has also contributed to the publication of a substantial number of studies based on spectroscopy. It is nowadays obvious that most laboratories and research centers have access to at least one kind of commercial spectroscopic instrument, due to the development and advancement achieved on these techniques and instruments, as well as relatively affordable prices.

Several recent studies have demonstrated the feasibility of using spectroscopic techniques for analyzing different thermal treatments in fish and seafood [4,7,17,18,19,20]. However, to date, no review has provided an integrated view of different cooking methods, which have already been in use or could be used in the future, along with traditional and new trends for evaluating and monitoring heat-induced changes provoked by thermal treatments. Therefore, this work reviews, for the first time, the application of spectroscopic techniques for monitoring thermal treatments of seafood in the last few years. The main objectives of this review are:

(i) To briefly present traditional cooking methods and recent advances in this context.
(ii) To discuss the impact of thermal treatments on different quality parameters of seafood.
(iii) To demonstrate the potential of spectroscopic techniques for monitoring thermal treatments rapidly and non-destructively in comparison with traditional methods.

## 2. Cooking Methods Used in Households and Seafood Industry

In modern society, fish is nearly always cooked prior to consumption. Cooking methods can be grouped into different categories according to several criteria (Table 1). Depending on the liquid involved, cooking methods can be categorized as moist (or wet) heat, dry heat, and combinations of dry and moist heat methods. Several cooking methods, such as boiling, steaming, roasting, baking, broiling, grilling, braising, frying/deep frying, etc., can be included within the previous categories. Cooking fish in water is one of the most traditional and popular cooking methods for fish or other food. Heat convection through water is the main form of heat transfer in such a low-temperature cooking method, and cooking in water can be called poaching (60–70 °C), simmering (80–90 °C), or boiling (100 °C), according to the water temperature [21]. Roasting is used to cook food at high temperatures, generally higher than 200 °C, while baking uses temperatures lower than 170 °C. Detailed information about cooking methods and their impact on food quality can be found elsewhere [22,23]. Uneven heat distribution and limited penetration depth are the main challenges related to the application of conventional heating methods in cooking food. The problem is even more challenging for fish and other seafood, due to their inhomogeneous composition and geometry. That is why a new trend has emerged in recent years, to avoid the negative consequences of the application of high temperature, thereby helping to retain most of the nutritional and sensorial quality attributes of treated products without compromising on safety. In this context, microwave, radiofrequency and ohmic heating are among the main emerging technologies that can be used instead of classical heat treatments. Besides, cooking with agitation (Shaka technology) and surface pasteurization have been investigated [13,24,25]. The main goal of these innovative technologies is to avoid unnecessary high heat load by reducing the overall thermal load (temperature/treatment time) and enhancing heat transfer to achieve a rapid heating. These new technologies will be briefly presented in this chapter without going into details about modelling of heat transfer and mass transfer during thermal treatments, as detailed information about this subject can be found in other review papers [26,27].

Microwave heating has gained great popularity in food processing due to its ability to achieve a high heating rate and rapid processing time. The electromagnetic waves generated in a microwave oven penetrate food and excite atoms of water and fat molecules, which start to vibrate and heat up the rest of the tissue. The principal issues with this technique are the unevenness of heating and the limited penetration depth, causing parts of the product to be underheated or overheated [28,29,30]. Radiofrequency heating achieves greater penetration depth, but also suffers from non-uniform distribution of heat and the need for direct contact between electrodes and samples [24]. Due to these drawbacks, the application of microwave and radiofrequency techniques at an industrial level is still limited to the thawing of food products, rather than heating. More detailed information about these dielectric heating methods (microwave and radiofrequency heating) in terms of concept and basic principles, as well as applications (alone or in combination with other techniques in food processing), can be found in other recent review papers [31,32,33,34].

Ohmic heating generates internal heat by passing an electrical current through the product, thus transforming the electric energy into thermal energy [12,35]. Previous reports indicated improvements, such as short heating time and reduced temperature gradients, in the quality of ohmic-heated products compared to traditionally-heated food [35,36]. Another strategy of minimal heat processing that can be used to reduce the heat load is the use of surface pasteurization, which aims at deactivating bacteria on the surface of fish muscle. This strategy is based on the hypothesis that the interior of the fish muscle is sterile immediately after slaughter, and that the surface of fish is the entry point of microbial contamination. Thus decontamination of the surface, by using a short time/high temperature heating process, might minimize the negative impacts of extensive heat processing [18,24,25,37]. However, a limited impact of the surface pasteurization on cod quality was reported in recent studies. Indeed, application of a surface pasteurization, with an equivalent of 62 °C at depth of 1 mm [37] or even 2 mm [25] below the muscle surface, was not sufficient to significantly extend the shelf life of treated samples. Agitated heat processing, or Shaka technology, is another effective strategy to enhance heat transfer rates, using reciprocal agitation instead of axial and end-over-end rotations. In contrast to traditional static autoclaves, the Shaka autoclave containing food is longitudinally agitated, which reduces heating time and saves significant quantities of energy. However, the technique is mostly applied for liquid and semi-liquid food, and documentation of process-safety, economy and heat transfer models are still needed [24,26].

Combined methods, or hurdle technology as it is termed in some references, refers to the combination of two or several preservation methods, which creates a cumulative or synergetic preservative effect. This combination strategy contributes to a better preservation of the sensory properties of treated food after heat treatment, leading to high-quality products, which is not readily achievable by traditional techniques or use of only one preservation method [1,12,38]. Several recently published studies have demonstrated that combining two or several preservation methods or strategies of thermal treatments can significantly improve the quality of treated products [28,33,38,39,40]. For example, recent studies showed that the combination of a water bath and microwave heating achieved better results, giving a surimi gel of higher quality than that heated with the traditional water bath alone [30,41].

According to the literature, there is no universal ideal method of cooking that can be applied to all fish or seafood products, since each method seems to have certain advantages and shortcomings. Although there is a substantial amount of data on the application of traditional thermal treatments and their impacts on quality, experimental studies and comprehensive characterization of some emerging techniques (e.g., agitated heat) are still lacking in the fish and other seafoods sector. Other recent techniques for minimal processing, such as high-pressure processing, pulsed electric field, ultrasound, and other non-thermal processes [3], should be investigated in future studies.

## 3. Monitoring Thermal Treatments

Depending on the heating method and the heating regime (i.e., sterilization, pasteurization, etc.), several changes in quality of fish can be induced during thermal treatments. These include changes in sensory attributes (e.g., texture, color, etc.), microbiological properties, physico-chemical parameters—such as modifications in proteins (e.g., denaturation, oxidation, etc.) and lipids (oxidation)—cooking loss, as well as changes in nutritional quality (e.g., digestibility). A wide range of traditional methods and spectroscopic techniques has been used to evaluate changes occurring in the quality of fish and other seafood during the application of thermal treatments.

### 3.1. Traditional Methods Used to Assess Changes in Quality Induced by Thermal Treatments

Texture, color, odor and other sensory properties make a significant contribution to fish acceptability by the consumers. Sensory parameters of fish and other seafood are modified when these products are subjected to heat processing, and the extent of these modifications depends mainly on temperature and duration of the treatment. A considerable number of studies have been carried out to investigate the impact of different thermal treatments on sensory properties, using a sensory panel and instrumental texture and color measurements [9,10,25,37]. Texture is often evaluated by the texture profile analysis (TPA) method, which simulates how samples behave while chewed by being compressed twice. Certain textural parameters, such as hardness, springiness, cohesiveness, gumminess, chewiness and resilience, are then obtained from force–time curves, representing plots of force as a function of measurement time. The TPA parameters (i.e., hardness, etc.) will not be detailed here, as they are discussed thoroughly in other publications [52,53]. Concerning the color, primary parameters, including *L∗* for lightness [the brightness of sample, ranging from black (0) to white (100), a* ranging from +60 (red) to −60 (green), and *b∗* ranging from +60 (yellow) to −60 (blue)], can be obtained directly by commercial laboratory instruments, while secondary (or derived) parameters, such as whiteness (the degree to which a sample surface is white), can be calculated from the primary parameters. Changes in microbial spoilage are often used along with the sensory analysis as a standard method to validate the shelf life of food products.

In addition to sensory and microbiological tests, several traditional physicochemical parameters, such as thiobarbituric acid reactive substances (TBARS) used as an indicator of lipid oxidation, total volatile bases nitrogen (TVB-N), water holding capacity (WHC) and cooking loss, are routinely monitored for evaluating quality modifications induced by thermal treatments. Other conventional measurements include proteins oxidation products, protein denaturation and formation of Schiff base/Maillard reaction products. Sodium dodecyl sulfate–polyacrylamide gel electrophoresis (SDS-PAGE) is a standard technique used to investigate changes in proteins following heat treatments [11,44]. Differential scanning calorimetry (DSC) has also been widely used as a reference method for measuring the thermal denaturation of proteins by calculating the difference between the amount of heat that is going into the sample and the amount of heat released from the sample [17,28]. Other techniques and approaches include scanning electron microscopy (SEM) to study heat-induced changes at microstructure levels of treated samples [39,54], enzymatic activity to follow degradation by heating of specific enzymes with known kinetics [55], and monitoring concentrations of certain pigments, which function as indicators of heat-induced color changes [56].

Examples of thermal processing methods commonly applied to fish and other seafood, and their impacts on quality parameters, are summarized in Table 1. Several studies have been carried out in order to optimize heat treatments in terms of time and temperature combinations, so that high levels of WHC and minimal liquid loss can be achieved during processing and storage [9,25,37,42,48,57]. For example, a wide range of processing times and temperatures, varying from 20 to 95 °C for 1 to 60 min, was investigated to optimize thermal treatments of cod (*Gadus morhua*), with respect to WHC and cooking loss, color and texture [9]. The authors reported that cooked cod muscles were harder, and their opaque color changed to white compared to raw samples, but no significant differences were observed between the cooked samples subjected to different thermal treatments. One of the main conclusions of this study was the possibility of keeping the WHC levels above 66% and cooking loss below 5.6% when cooking the fish at approximately 68 ± 4 °C for 25–35 min. More recent studies investigated the impact of surface pasteurization on the sensory and microbiological quality of cod fillets heated at various temperatures and times [25,37]. Among other results, these studies revelated the challenge of directly linking the spoilage rate of heat-processed foods with the applied thermal load. Indeed, contradictory to what was expected, the authors detected more microbial growth in the cod samples which had lower contamination before the heat processing and were subjected to more severe heat treatment. Llave and colleagues investigated the impact of cooking tuna, at a low temperature (59 °C) with two cooking times (cooking for 39 min versus 13 min), on thermal denaturation and the following impacts on texture and color [58]. A strong influence of the cooking method was observed on quality parameters, which was attributed to thermal protein denaturation, especially denaturation of myosin which was reported to be the main agent responsible for changes in the color of samples, while denaturation of actin had a significant impact on changes in the texture. In addition, the authors observed that the samples cooked during a short time had better quality attributes in terms of taste and juiciness.

The impacts of thermal treatments on sensory quality, microbiological properties and physico-chemical parameters of fish quality have been well documented in the literature. However, little research has been conducted to investigate the effects of different cooking methods and heating regimes on the nutritional quality of these products. It is only in very recent years that some studies have been conducted on this topic [10,11,44]. Protein digestibility is one of the essential factors that determines the quality of proteins and their assimilation, as well as the accessibility and bioavailability of amino acids. In a recent study [10], three different cooking methods (boiling, baking and frying) of hairtail (*Thichiurus lepturus*) fillets were investigated with respect to their impact on protein modification and digestibility. The results showed that the frying method greatly affected nutritional quality, since lower in vitro digestibility levels were observed for fried fillets compared to the boiled and the baked samples, while higher acceptability of hardness and color was obtained for baked fillets compared with the two other cooking methods.

Several drawbacks are often associated with the aforementioned conventional methods. In addition to being offline, most of these techniques are laborious, time-consuming, and on the top of that, these methods cannot be used to monitor the whole processed product, as they are sample-destructive. Thus, in the industrial environment, the whole production volume is not scanned, but only a few samples, which are chosen on a random sampling basis, are actually used for testing. These samples should perfectly represent the complete product volume, otherwise a large uncertainty of measurement will be introduced [7]. However, food sample matrices are very complex, and it can be difficult to assure that a few samples can represent the whole production volume. This is especially the case for seafood products, as a considerable variability between species, and even from fish to fish within the same species, is commonly observed. Moreover, in some cases, such as ready-to-eat products consumed without further heating, all the production should be monitored, not just selecting some random samples [16]. Thus, continuous and real-time control using non-destructive methods, such as spectroscopic techniques, are safer and more adequate for the modern seafood industry.

### 3.2. Monitoring Thermal Treatments by Spectroscopic Techniques

**Figure 2:**
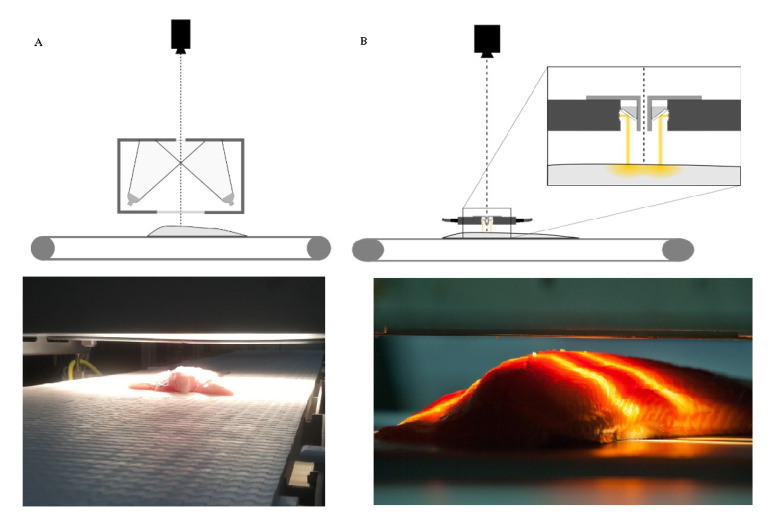
Hyperspectral imaging setups used to scan samples on conveyer belts with (**A**) diffuse reflectance mode and (**B**) interactance mode.

Over the past several decades, various spectroscopic techniques, such as infrared (IR) (including mid-infrared, MIR; and near-infrared, NIR), Raman spectroscopy, nuclear magnetic resonance (NMR), and fluorescence spectroscopy, have been widely used in many applications in order to address the aforementioned challenges of the traditional techniques. Depending on the position of the illumination source, the scanned sample and the detector, measurements can be performed using various modes; reflectance/diffuse reflectance, transmission and interactance. In reflectance/diffuse reflectance modes [59], the detector and the light source are placed above the sample on the same side, while in the case of the transmittance mode, the detector and the light source are placed on opposite sides of the sample. In the interactance mode [60], the sample is illuminated and measured on the same side, but the illumination is focused on an area adjacent and parallel to the field of view of the detector (Figure 2).

**Figure 3:**
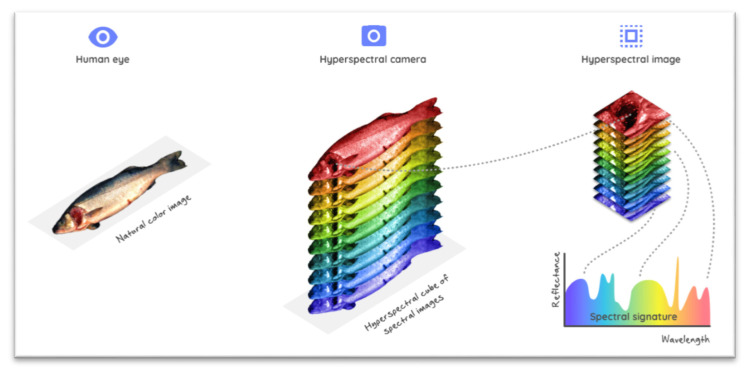
Hyperspectral data cube (hypercube) viewed as either an image at each individual wavelength or as a whole spectrum at each individual pixel of the image.

More recently, hyperspectral imaging (HSI) techniques have emerged as efficient and promising analytical tools in many important application areas. The techniques combine imaging and spectroscopy in a single system, thus providing information about internal (chemical composition) and external (physical features) attributes simultaneously [61,62]. In other words, HSI combines the merits of traditional imaging and spectroscopic techniques at the same time, thus enabling the acquisition of simultaneous spatial and spectral information from the examined samples, which is especially useful for non-homogeneous samples, like fish and seafood products. By using HSI, each pixel of the image contains high-resolution information on light intensity as a function of wavelength. This means that HSI outperforms color cameras, which register light intensity only in three broad wavelength ranges (red, green and blue). Data generated from HSI, called hypercubes, have a three-dimensional structure (x, y, *λ*), corresponding to two spatial dimensions (x rows × y columns) and one spectral dimension (comprising a range of wavelengths *λ*). The results obtained from HSI can be seen either as a separate spatial image (x, y) at each individual wavelength (*λ*), or as a whole spectrum (*λ*) at each individual pixel (x, y) (Figure 3). More interestingly, by using appropriate multivariate calibration models, every pixel of the hyperspectral images can be transferred into chemical images, displayed in a linear color scale with different colors. These chemical images visualize quantitative spatial distributions of the predicted examined parameters along the tested fish sample, and their corresponding concentrations.

More detailed information about the fundamentals and recent advances in spectroscopic techniques have been well documented elsewhere [14,61,63,64]. Thus, the current review will focus only on the application of these techniques for monitoring thermal treatments in fish and other seafood products.

Spectroscopic techniques should be combined with suitable multivariate data analysis, as the measurements usually generate a huge data size, especially if the spectroscopic methods are coupled with HSI. Several spectroscopic methods have been used, in order to classify samples on the basis of quality changes occurring during thermal treatments, or to predict quality parameters of samples treated under different cooking methods or cooking conditions. Therefore, a short description of multivariate data analysis, including mainly classification and prediction techniques, is given here.

Class modelling and discriminant analysis have been used in numerous studies to classify samples according to cooking methods or cooking conditions. Partial least squares discriminant analysis (PLS-DA), linear discriminant analysis (LDA) and support vector machine (SVM) are among the most-used classification models [4,65]. Regarding the prediction, partial least squares regression (PLSR) is a linear model that has been most commonly used. However, more complex nonlinear models, such as artificial neural network (ANN) and support vector machine (SVM), have gained more attention in recent years. The quality of these models is generally evaluated by calculating determination coefficients (R^2^) and the corresponding root mean square errors (RMSE) for both calibration and prediction datasets. It should be mentioned that, usually, dimensionality reduction methods, such as principal component analysis (PCA), among others, are commonly used before resorting to classification and prediction models.

#### 3.2.1. Visible and Infrared Spectroscopy

Absorbance in the ultraviolet (250–400 nm) and the visible (VIS) regions (400–780 nm) results from electronic transitions, while overtones and combinations of vibrational frequencies are responsible for signals in the NIR region (780–2500 nm). MIR spectroscopy is associated with fundamental vibrations and provides a greater amount of chemical information than NIR spectroscopy [14,66].

HSI in the NIR wavelength range has been used in several research investigations to study quality attributes in fish and other seafood that are affected by thermal treatments. In one of these studies [4], an HSI system was employed in the spectral range 950–2400 nm to monitor changes in core temperature and to predict the thermal history of kamaboko, a kind of Japanese seafood. The results showed a good performance of the technique in both classification and prediction modelling. Indeed, a classification accuracy of 93.8% was obtained by applying the LDA model, and a reasonable fit, with R^2^ of 0.86 and 0.83 for, respectively, core temperature and thermal history, was obtained by applying the PLSR model. In another study, VIS/NIR spectroscopy was proposed to predict the endpoint temperature of Alaska pollock surimi heated in a water bath for 2 min at a controlled temperature, varying from 45 to 90 °C [19]. The authors observed a decrease in absorbance with increasing cooking temperature, which was attributed to changes in scattering caused by protein denaturation. Promising results with low RMSE and high R^2^ were obtained in the visible spectral range (i.e., 400–700 nm), due to the low absorbance of water in this spectral range compared to the NIR region. In a similar approach, one batch of surimi was cooked in a water bath at different temperatures (ranging from 70 to 95 °C) and cooking times (2–30 s), and another batch was heated in steam, and the heat transfer process was evaluated using VIS/NIR spectroscopy [18]. Different kinetics were observed from the spectroscopic responses obtained by the two cooking methods, and again, the visible range gave the best results. However, it should be stressed that the results in the two previous studies were based on static offline measurements under laboratory conditions, which may not be representative of real industrial applications.

More recently, two different instruments based on NIR (760–1040 nm), namely a point measurement system and an HSI scanner, were evaluated for their potential as non-destructive tools for determining the core temperature in heat-treated fish cakes [7]. The main advantage of this investigation is that the author evaluated the performance of both instruments in measuring the same product in a static mode (offline) and on a moving conveyor belt (inline), thus mimicking a typical industrial environment. The point measurement system performed better than the imaging system, with RMSE of 2.3 °C and 4.5 °C for the point measurement and the imaging system, respectively. The difference in performance between the two instruments was explained by higher exposure time—thus less noisy signal—and the higher optical penetration depth of the point system compared to the imaging scanner.

Over the past few decades, development of IR instrumentation has led to a more efficient technique called FTIR, based on the implementation of Fourier Transform (FT) in IR spectroscopy. This technique has many advantages over the traditional IR technique, such as acquisition of data from solid, liquid or gas samples, with high spectral resolution over a wide spectral range. The potential of FTIR spectroscopy has been well documented for various applications, and the technique has been applied in many different fields. Several studies have suggested the use of FTIR spectroscopy—mainly in the spectral region 1600–1700 cm^−1^, referring to the amide I band—as a useful tool for studying protein secondary structures as affected by thermal processing (Table 2). For example, FTIR was proposed as a suitable method for monitoring changes in the protein denaturation of Atlantic salmon (*Salmo salar*) during thermal pasteurization [6]. In this study, PCA was applied to study the variance in spectra of the samples heated at different temperatures and heating times. With increasing time and temperature, the intensity of the peak related to the α-helix decreased, while that related to the β-sheet peak increased, indicating an increase in protein denaturation and protein aggregation, respectively. FTIR results were in accord with those of DSC, indicating that the myosin and sarcoplasmic proteins were found to be completely denatured after 20 min at 55 °C, after 4 min at 65 °C, and after 1 min at 75 °C, and were not detectable at 85 and 90 °C. These results were in agreement with those obtained in a previous study [40], which also showed a decrease in α-helix structure and an increase in β-sheet structure contents with increasing heat loads. In another study, gel strength, as one of the most important quality indicators for surimi products, was evaluated by FTIR spectroscopy after heating Alaska pollock surimi at high temperatures, namely above 100 °C for 10 min [67]. By analyzing the amid I region, the authors noticed a significant decrease in random coil and other protein secondary structures with increasing treatment temperature, indicating protein aggregation and polymerization and a reduction in the gel strength. In addition, based on the results obtained regarding the texture, WHC and the volatile compounds, the authors suggested heating the surimi at 110 °C to get the best results.

#### 3.2.2. Raman Spectroscopy

Similar to IR spectroscopy, Raman spectroscopy is also a vibrational technique that can be used to study protein conformation and structural changes during processing, but the technique does not suffer from water absorption as does IR spectroscopy, because Raman is based on measuring inelastic light scattering. Although considered a very promising tool for quality analysis due to its non-invasive and rapid detection, Raman spectroscopy is often used as a complementary tool to infrared absorbance [40,72], and little research has been conducted to assess the potential of this technique for monitoring thermal treatments (Table 2).

For example, protein structural changes occurring in white shrimp during heat treatment at 50 °C for 10–30 min were investigated using Raman spectroscopy [68]. Similar to the FTIR results, the amid I region, and more precisely the *α*-helix and β-sheet structures, showed significant modifications as a result of protein denaturation induced by the heat treatment. In more detail, the amid I band maximum was shifted toward high frequencies from raw fresh shrimp to heated shrimp, especially for samples heated for longer times. In addition, changes in physical properties, including texture parameters and water loss, were observed in the shrimp samples heated for different times. In another study [40], it was demonstrated that Raman spectra obtained in the range 300–1800 cm^−1^ on hairtail surimi can be used to study structural changes caused by irradiation and heat treatments. Indeed, a decreased content of the α-helix and an increased content of the β-sheet were observed with increasing temperature, suggesting the usefulness of Raman spectroscopy for monitoring the secondary structure of protein in surimi during thermal processing.

#### 3.2.3. NMR Spectroscopy

Recently, low field nuclear magnetic resonance (LF-NMR) and magnetic resonance imaging (MRI) have gained more interest, and applications have increased during the last few years, due to the development of benchtop spectrometers which are compact, robust and affordable. For more detailed information about NMR instrumentation, fundamentals and principles, the reader is referred to other references [73,74].

Some of the applications of NMR in monitoring thermal treatments of seafood are summarized in Table 2. Recently, NMR spin–spin relaxation (T_2_), or the transverse relaxation, was investigated to study water distribution and migration within bighead carp (*Aristichthys Nobilis*) gel, treated at temperatures varying from 40 to 90 °C for 30 min [54]. Using PCA, the study provided useful information for further understanding the relationships between NMR-measured parameters and water mobility, in relation to different water holding mechanisms, gel microstructure and myosin secondary structure, during the heating process at various temperatures. In addition, the SEM micrographs showed significant differences in the physical structure of gels as a function of heating temperature. In a recent study [49], the impact of cooking time (from 5 to 60 min) during boiling treatment (at 95–100 °C) of false abalone (*Volutharpa ampullacea perryi*) was investigated using NMR and MRI techniques. NMR results displayed a reduction in immobilized water with increasing cooking time, which was accompanied by a decrease in the shear force and sensory acceptability of this seafood product. In accordance with the NMR results, the T_2_ weighted images from MRI showed a decreased amount of free water and an increased amount of bound water in the fish with increasing heating time. In a similar and more extensive study, three cooking methods (frying, boiling and stewing) of turbot were assessed by the relaxometry method of NMR and MRI, and the measurements were correlated with quality parameters of texture and color [53]. Different relaxation times were identified for the different cooking methods, indicating different water dynamics according to the heat treatment method. PCA applied to the NMR data showed a clear discrimination between the samples according to the cooking method. Moreover, weighted images of the MRI scans provided visualizations of internal structural information, which confirmed the results obtained from the NMR measurements.

#### 3.2.4. Fluorescence Spectroscopy

Fluorescence spectroscopy is one of the most attractive spectroscopic techniques due to its high sensitivity and high selectivity, which outperform most traditional spectroscopic techniques [75,76]. Fluorescence refers to the light emitted from samples after excitation of specific molecules called fluorophores. Fish and other seafood naturally contain several compounds that can act as fluorophores, such as nicotinamide adenine dinucleotide (NADH), vitamin A, riboflavin, aromatic amino acids, lipid oxidation products, collagen, and many other molecules. Changes in fluorescence properties as a function of cooking methods and cooking conditions have been widely investigated in several food matrices, such as meat [77,78,79], milk [80,81], liquid butter [82] and oil [83], among others. However, little or no research has been found in the literature regarding fish and other seafood products. Therefore, future research should investigate possible relationships between changes induced by thermal treatments and fluorescence spectra obtained from samples subjected to various cooking methods and different heating conditions.

Some recent studies have used fluorescence spectroscopy as a complementary tool to support results obtained from other traditional measurements (Table 2). For example, this technique was used as an indicator of the formation and progression of Maillard reaction products in hairtail fillets subjected to three different cooking methods, namely boiling, baking and frying [71]. Maillard reaction is known to occur during the thermal processing of food at high temperatures and plays the role of precursor to a wide range of aroma and color compounds. The authors measured fluorescence emission at 415 nm after excitation at 347 nm for raw and cooked fish fillets, and the result indicated much higher fluorescence intensity in the baked and fried samples compared to the raw and boiled ones. The traditional measurements showed that the boiled samples contained lower protein and fat, but better maintained their lightness and generated very low levels of Maillard reaction products when compared to the baked and fried samples. In another study, the impacts of five cooking methods (i.e., roasting at 200 °C for 10 min, frying at 180 °C for 5 min, boiling and steaming at 100 °C for 8 min, and microwaving at 800 W for 6 min) of farmed sturgeon (*Acipenser gueldenstaedtii*) on protein and lipid oxidation, SDS-PAGE and the formation of fluorescence compounds were investigated [44]. Emission spectra obtained after excitation at 360 nm, referring to Schiff-base structures formed during cooking as a result of interactions between myofibrillar proteins and lipids, displayed various patterns as a function of cooking methods. Indeed, the fluorescence intensity obtained from samples heated by the extreme cooking methods, namely roasting and frying, was higher, thus indicating greater formation of Schiff-base structures compared to samples heated with the lighter cooking methods (i.e., boiling, steaming and microwaving). Except for the roasted samples, significantly higher values of TBARS were observed in all cooked samples compared to raw fish. In addition, the content of carbonyl groups was around four times higher in the fried and roasted samples than in the raw fish, indicating a more harmful effect of these two cooking methods compared to cooking in water. Moreover, the SDS-PAGE profiles demonstrated that the myosin heavy chain (MHC) was the most sensitive protein to the type of cooking method, with the intensity of this protein band being significantly decreased in the fried and roasted fillets compared to the microwave-cooked, steamed and boiled samples. In a similar study [11], the same authors used fluorescence spectroscopy with excitation at 360 nm to study the impact of roasting time on formation of lipid and protein oxidation products during heating of sturgeon samples at 200 °C. An increase in fluorescence intensity was observed with increasing cooking time, which was attributed to the accumulation of Schiff-base structures and the formation of carbonyl derivatives. During the first 5 min of roasting, the digestibility level increased due to the protein unfolding, which allows pepsin to access more active sites, while longer heating time induced aggregation, polymerization and the formation of cross links, thus reducing the digestibility. Additionally, the authors noticed from the SDS-PAGE profiles a decrease in the intensity of most of the protein bands of the roasted samples with increased roasting time, which was explained via loss of solubility due to protein denaturation and breakdown of large proteins during heat treatment. In a recent study [17], a close relationship between, on the one hand, hardness and total collagen contents, and on the other hand fluorescence intensity, was shown in Atlantic mackerel (*Scomber scombrus*) fillets cooked in a sous-vide water bath. From the endothermic peaks obtained from the DSC profile, it was shown that myosin started degrading at a low temperature (28 °C), while peaks located around 32, 49 and 68 °C were attributed to the shrinkage of collagen, gelation of collagen and denaturation of actin, respectively. In a more recent study, the same authors demonstrated a linear correlation between fluorescence intensity, obtained at 530 nm with excitation at 470 nm, and the oxidation products formed in sous-vide-cooked mackerel samples with increasing cooking time and cooking temperature [20]. In the previous two works, the authors used fluorescence in microscopic images, and the resulting fluorescence micrographs enabled the visualization of the molecular evolution of collagen fibrils and oxidation products during different cooking conditions. 

It should be stressed that most of the aforementioned fluorescence studies have been conducted offline as laboratory applications, which is regarded as hardly compatible with industrial environments. In addition, univariate analysis (Table 2) applied in these studies may not be an appropriate way to describe complex reactions occurring during the application of thermal treatments to complex food matrices, like fish and other seafood. Besides, characterization and assignment of the observed fluorescence to specific fluorophores is a difficult task, especially when using a single excitation wavelength, as was the case in the previous studies. Thus, further future studies are required in order to identify the most effective and relevant excitation/emission wavelengths, and the corresponding fluorophores, according to different processing conditions. This is possible by using fluorescence excitation–emission matrices (EEM) instead of traditional fluorescence spectroscopy, which records fluorescence spectra at only a single excitation wavelength. The EEM mode measures over a whole range of emission spectra at different excitation wavelengths, creating a three-dimensional map for excitation, emission and response, covering the total fluorescence response.

**Figure 4:**
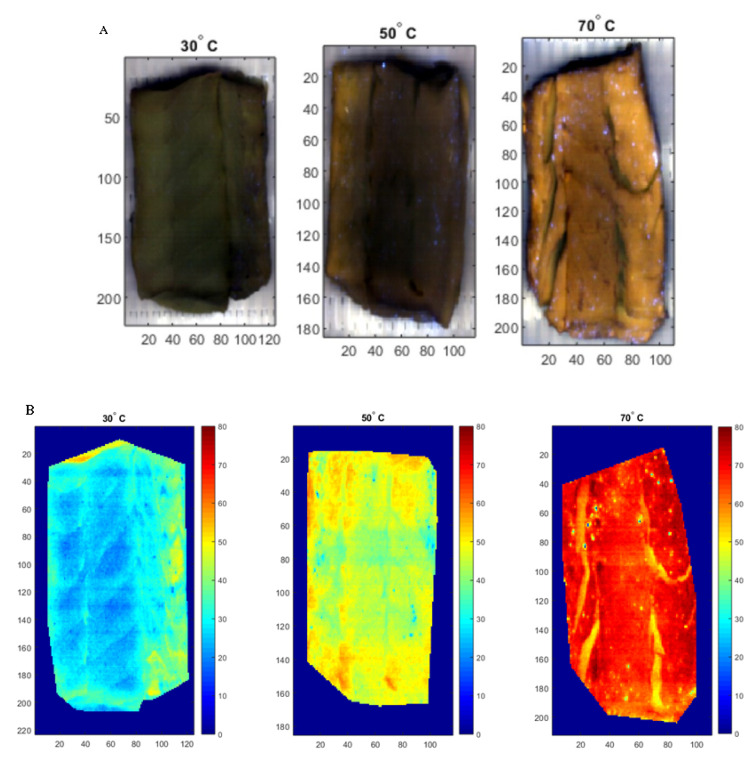
Examples of pseudo green red blue (RGB) images of cod fillets cooked at three different temperatures (30, 50, and 70 °C) and measured by fluorescence hyperspectral imaging (**A**); Pixel-wise estimates of temperature, visualizing the temperature distribution in the fillets (**B**).

Another observation from the reviewed literature is that HSI has been mostly used with the VIS and NIR ranges, operating in interactance and reflectance modes, while very little information is available on the use of HSI in the fluorescence mode [15,84]. The combination of fluorescence spectroscopy, as a highly sensitive and selective technique, and HSI offers a unique possibility for full control and optimal monitoring of thermal treatments. Therefore, our research team has investigated the potential of fluorescence HSI, performing online in a continuous mode with a conveyor belt, for monitoring cooking regimes in packed cod fillets processed via sous-vide at three temperatures (30, 50, and 70 °C). A decrease of fluorescence intensity was observed with increased cooking temperature, probably due to a decrease in collagen content and a degradation of connective tissue. The PLSR model was used to process hyperspectral fluorescence imaging data, and the model was applied to the images in order to show temperature distribution spatially for each pixel. A linear color bar, ranging from blue to red, was used to display variations in the predicted temperature (Figure 4). It can be easily observed that there was a general trend in color, shifting from blue to yellow and then to red, with the increase in temperature, suggesting that changes in cooking temperature can be monitored using fluorescence HSI data. In another study, we demonstrated the potential of fluorescence spectroscopy for the online monitoring of thermal treatments applied to cod fillets cooked sous-vide in a water bath [65]. In this study, both linear (PLS) and nonlinear (SVM) prediction and classification models displayed good performances in predicting cooking temperature and classifying the cod samples according to their cooking temperature.

## 4. Limitations and Future Trends

From the information published in the literature in the last few years, it can be demonstrated that spectroscopic techniques coupled with chemometric tools have a strong potential use in the assessment of thermal treatments of fish and other seafood. Due to their high speed, relatively low cost and, most importantly, the possibility of application online/inline in an industrial environment, these techniques can be used as alternatives to sample-destructive, time-consuming and offline methods. Moreover, most conventional methods are used for monitoring only one quality attribute (such as TBARS, hardness, etc.), while spectroscopic techniques are non-targeted fingerprint approaches, providing several quality parameters at the same time. This allows unique, detailed profiles and complex chemical information to be obtained from the examined samples. Besides, spectroscopic methods allow the scanning of every single sample, instead of choosing only a few random samples as representative of the whole production batch, as is the case currently when performing traditional measurements.

The application of traditional cooking methods with severe heat can result in degradation of nutritional quality and changes in sensory and other quality attributes. Understanding the mechanisms of quality changes in seafood products subjected to thermal treatments is key for the further optimization of heating processes and quality control in these products. Several quality modifications, such as protein denaturation, lipid and protein oxidation, and changes in texture, color and other sensory and physico-chemical attributes, can be evaluated via spectroscopic methods, and used as indicators for monitoring the thermal treatment of seafood products. Application of spectroscopic techniques for evaluating or predicting some of these changes has already been explored and validated in previous investigations. For example, vibrational spectroscopic techniques, especially MIR and Raman, have been successfully used in many studies in order to investigate changes in the secondary structure of proteins induced by the application of different thermal treatments. NIR has a broader non-destructive usage scope and has been examined to monitor the core temperature of heated products, particularly when used in the interactance mode, which allows light to travel deeper into the sample. Color and other external quality parameters can be predicted using the diffuse reflectance mode, while internal quality parameters and chemical attributes require application of the interactance mode. Given the capability of NMR to monitor the behavior of water and its distribution in fish muscle, as well as its relationship with structural changes occurring in the cooked product, NMR measurements can be used to evaluate the extent and severity of the thermal treatment to which the fish or other seafood was subjected. Fluorescence spectroscopy has been reported in several studies to be the most sensitive and selective technique among the different spectroscopic methods. This technique has shown great promise in the estimation of oxidation products, protein denaturation, changes in collagen, etc., that take place during application of thermal treatments. Nevertheless, to date, the technique has been mainly used as a complementary tool to support other results obtained from traditional measurements, such as measurements of the formation of Maillard reaction products and Schiff bases, among others.

However, changes in other quality parameters, such as microbiological parameters and nutritional quality, resulting from applications of thermal treatments remain to be studied in future spectroscopic work.

From an analytical point of view, some challenges still need to be overcome. One of the main difficulties in monitoring thermal treatments in aquatic products is the considerable natural and inherent biovariability between species, and even between fish within the same species. Indeed, not only the length and weight of fish, which vary with age, state of maturity and nutritional status, but also many other factors, such as fishing area, season, fishing techniques, etc., could affect the raw material used before thermal processing. In addition, in the fish body itself, there is a certain degree of variability between the different parts of the fish; for example, the distribution of water, protein and fat is not even along the fish fillet, and can vary from the head to the tail and among the ventral and dorsal parts. Moreover, the inhomogeneous shape of fish can create additional analytical challenges. For instance, it can be difficult to maintain the same distance between the irregular-shaped sample and the detector. Therefore, careful considerations should be taken before drawing specific conclusions. For example, a difference in spectra obtained from heat-treated samples can be due to the variability in the raw material before processing, rather than the thermal treatment itself.

To address the challenges related to monitoring quality in inhomogeneous food products such as fish, a combination of several imaging modes, such as the interactance mode for measuring internal parameters and the diffuse reflectance mode for detecting external features, or even a combination of several spectroscopic techniques, such as vibrational (NIR, MIR, Raman) and fluorescence spectroscopies, could be a promising alternative. This strategy, of the application of different measurement techniques conducted on the same sample, assumes that different types of information obtained using different techniques can be complementary to each other, thus enhancing the obtained results. Although this combination approach, called hurdle technology, is well-known and well-documented in the literature regarding processing methods (such as the application of heat processing combined with vacuum-packaging), little research has been conducted on combining two or several analytical methods. Therefore, the hurdle concept should be extended to include analytical methods used for monitoring thermal treatments, since heating is a complex process, involving several chemical reactions and various changes in quality parameters. Another strategy to overcome the heterogeneity issue of samples is to combine the aforementioned techniques with HSI, which enables spectral and spatial information to be obtained simultaneously. Our review of the literature showed, however, that most of the published studies have been conducted focusing only on the spectral features, while the spatial features have been almost ignored. Thus, future studies should be aimed at enhancing the performance of HSI by investigating the joint features of spectral and spatial characteristics in combination with fluorescence spectroscopy.

Currently, most of the applications of spectroscopic techniques for monitoring thermal treatments appear to be research-oriented, under controlled and static laboratory conditions. While most studies conducted have pretended to have online applications, a closer examination revealed that only a few applications have been developed with moving samples on conveyor belts to mimic an industrial environment. However, the high need and demand of consumers and the seafood industry for routine and real-time monitoring has become a driving force, stimulating greater effort and more intensive research into the transfer of such laboratory-based measurements into online industrial processes. This includes the transfer from laboratory bench-top applications to a pilot scale and semi-industrial environment (under conditions close to industrial applications) using prototypes, and then to an application under real practical conditions. The process should be accompanied by improvement in computing power and speed, as well as increases in camera resolution, thereby enabling the acquisition of better images quickly and in real-time.

Another analytical observation derived from our review of the literature regards the scarcity of applications and relevant research data on thermal treatments using fluorescence spectroscopy. Despite the high specificity and sensitivity of this technique, which makes it an effective tool for monitoring thermal treatments, little work has been found in the literature. Moreover, most studies have been conducted with measurements at only a single excitation wavelength, which limits the ability to follow the several fluorophores that might be present in samples, especially in complex multifluorophoric mixtures, such as fish. To address this challenge, use of fluorescence with the EEM measurement mode should be further investigated in future applications. This method allows the acquisition of fluorescence data in three dimensions, with a wide range of excitation and emission spectra at the same time. However, fluorescence spectroscopy still suffers from some challenges, including restriction of the techniques to fluorescent compounds (fluorophores), the difficulty in attributing fluorescence properties to specific fluorophores, the possibility for overlapping signals from different fluorophores, and an inner filter effect resulting from absorption of the excited and emitted radiation. Furthermore, scanning a whole EEM is time-consuming, even though recent advances in new instrumentations allow one to significantly reduce acquisition time. By addressing these challenges, it is believed that a combination of HSI with EEM fluorescence spectroscopy can lead to a real revolution in the monitoring and optimization process in many seafood applications.

From a technological point of view, our review of the literature shows that there is no ideal cooking method for all seafoods, as each technique has its own advantages and disadvantages. Several studies indicated that heating under high-temperature methods (such as deep-frying) or the application of high thermal load can make seafood susceptible to a loss of nutritional value. The application of mild thermal processing, through the combination of two or more traditional techniques or the use of innovative techniques, seems like a promising solution to reducing the negative consequences of high thermal loads. Some of these innovative processing techniques have shown promising results in other food products, but their applications are still lacking in the seafood sector. Existing cooking methods, such as dielectric heating techniques (e.g., microwave and radio-frequency heating), should be optimized, and innovative methods (e.g., Shaka technology) should be tested further on fish and other seafood in order to enable smarter, healthier and faster cooking. 

Although the effect of heat treatments on the sensory, microbiological and physico-chemical parameters of fish and other seafood has been well-documented, there is still a lack of information in the literature regarding the influence of different cooking methods on nutritional quality. Only a few studies have recently reported changes in protein digestibility in fish products based on thermal treatments. Hence, more studies should be conducted in the future in this area.

## 5. Conclusions

The studies discussed in the current review have demonstrated that thermal treatments can be monitored via several spectroscopic techniques, such as infrared, NMR, Raman and fluorescence spectroscopy, and that the results can be further enhanced by coupling these techniques with HSI. However, some challenges need to be addressed, with intensive research and more effort being directed towards more practical and industrial environments, before it is possible to achieve a transfer of laboratory-based, offline measurements to online/inline processing control. Thus, several applications in both thermal process optimization and quality monitoring remain to be explored in more detail with more intensive studies. In order to increase the efficiency of thermal processing and to enhance seafood quality, innovative techniques should be developed, and the hurdle concept should be applied with both emerging processing technologies and analytical techniques. With further developments and improvements, it is expected that spectroscopic techniques will successfully carry out different tasks and find wider applications in the seafood industry in the near future.

## Figures and Tables

**Table 1:** Summary of some relevant research results regarding the commonly used cooking methods and their impacts on quality parameters of fish and other seafood.

Fish or Other Seafood	Cooking Method (s)	Quality Parameter (s)	Main Results	References
Hairtail	Boiling, baking, and frying	Nutritional quality: digestibility	Boiled fillets exhibited higher digestibility compared to the fillets cooked by baking or frying	Tavares et al. 2018 [10]
Salmon	Heating in an oil bath	Physico-chemical parameters: cooking loss, area shrinkage, color, texture	Most of the cooking loss and area shrinkage occurred within the first half hour of cooking, then approached an equilibrium. Color tended to be whiter, then browner, and texture gradually became soft as heating progressed	Kong et al. 2007 [42]
Blue mussel	Heating in an oil bath	Physico-chemical parameters: cooking loss, protein shrinkage, and texture	Cooking loss increased with increasing cooking time and temperature and correlated positively with area shrinkage and compression force	Ovissipour et al. 2013 [43]
Sea bream surimi	Microwave and water bath	Physico-chemical parameters: gel strength, WHC, microstructure morphology	Microwave- and water bath-treated samples demonstrated better gel properties (stronger gel with finer texture and increased WHC) than samples heated only with microwave	Cao et al. 2018 [39]
Sea bream surimi	Microwave and water bath	Gel texture properties, dielectric properties, WHC, cooking loss, color	Surimi paste with a thickness of 2 cm of surimi gave the highest WHC, lowest cooking loss, uniformity of temperature distribution, higher tensile force and whiteness	Cao et al. 2019 [30]
Sturgeon	Boiling, steaming, microwaving, roasting, and deep-frying	Physico-chemical parameters: protein (carbonyls, Schiff bases, and free thiols) and lipid (TBARS) oxidation	Higher proteins and lipid oxidation in roasted and fried samples than those cooked under less intense cooking conditions (boiling, steaming and microwaving)	Hu et al. 2017 [44]
Atlantic salmon	Microwave and conventional pasteurization	Sensory, microbial growth, protein denaturation, color, and liquid loss	Combination of CO_2_ with heating increased shelf life compared to heating (with microwave or conventional pasteurization) without presence of CO_2_ in vacuum-package	Lerfall et al. 2018 [28]
Shrimp	Water bath	Cooking loss, shrinkage, texture, and color	Hardness, cooking loss, area shrinkage, and overall color change increased with thermal load	Wang et al. 2018 [45]
Grass carp	Microwave	Denaturation and aggregation of sarcoplasmic and myofibrillar proteins	Increased microwave power and treatment time induced a decrease in solubility and an increase in turbidity, indicating a more aggregation of proteins	Cai et al. 2017 [46]
Atlantic cod	Water bath	Microbial and sensory analysis, pH, and liquid loss	Mild heat treatment with high temperature and short cooking time decreased liquid loss and inactivated bacteria of the muscle surface, but did not extend shelf life significantly compared to samples cooked at lower temperature for longer time	Stormo et al. 2018 [19]
Fish patties: silver smelt	Water bath	Microbial growth, water content, lipid content, pH	Deceased bacterial load with increasing cooking temperature and time. Increased shelf life for samples packed with high CO_2_ levels compared to regular modified atmosphere packaging or vacuum	Abel et al. 2019 [47]
Cod	Convection oven	Rheological analysis, WHC, mass loss	Development of a model to predict temperature and moisture concentration based on both heat and mass transfer during cooking	Blikra et al. 2019 [48]
False abalone	Boiling	Sensory, texture properties, protein denaturation, water distribution	Increasing cooking time resulted in a decrease in shear force and sensory acceptability, with a rapid denaturation of proteins (denaturation at the first minute). Development a model to predict myosin and actin denaturation	He et al. 2018 [49]
European sea bass	Microwave and conventional oven	Lipid content and composition, and volatile compounds	Conventional oven backing gave higher abundances of volatile compounds than microwave cooking and salt-crusted oven baking	Nieva-Echevarría et al. 2018 [50]
Atlantic cod	Water bath	Cooking loss, WHC, color, and texture	Optimal cooking conditions by cooking with heat that is equivalent to 2 min at 70 °C, thus keeping cooking loss below 5.6% and WHC above 66%, along with low hardness and whiteness values	Skipnes et al. 2011 [9]
Atlantic Mackerel	Water bath	Protein solubility, lipid oxidation, color, texture, cooking loss, and pH	Increase in lipid oxidation products, lightness, yellowness with increasing storage time. Liquid loss increased with increasing cooking temperature but decreased with storage time. Heating caused a reduction in protein solubility	Cropotova et al. 2019 [51]

WHC, Water holding capacity; TBARS, Thiobarbituric acid reactive substances.

**Table 2:** Examples of applications of spectroscopic techniques for monitoring thermal treatments applied to fish and other seafood.

Fish or Other Seafood	Applied Technique	Wavelength or Wavenumber Range	Model	Key Issues-Outcomes	References
Walleye pollack and horse mackerel gel	VIS/NIR	650–1100 nm	PLSR, MLR	High accuracy of prediction of heating temperature (R = 0.98 with prediction error of 1.85 °C). Spectral changes were attributed to protein denaturation and changes in the state of water, induced by the heat	Uddin et al. 2006 [67]
Kamaboko	NIR	900–2500 nm	PLSR, LAD	Reasonable level of accuracy for prediction of core temperature (R^2^ = 0.86, with prediction error 3.9 °C) and thermal history (R^2^ = 0.83 with prediction error 0.29 min) and high classification accuracy	Elmasry et al. 2015 [4]
Surimi	VIS/NIR	400–2500 nm	PLSR	Best results for prediction of cooking surface temperature in the visible range 400–550 nm (R^2^ ≥ 0.9) with prediction errors lower than 3 °C	Skåra et al. 2014 [18]
Surimi	VIS/NIR	400–2500 nm	PLSR	Endpoint temperature was best predicted in the visible range 400–700 nm (R^2^ = 0.96) with a small prediction error (<2 °C)	Stormo et al. 2012 [19]
Fish cakes	NIR	760–1040 nm	PLSR	Prediction core temperature in the fish cakes with prediction errors of 2.3 °C (NIR point system) and 4.5 °C (imaging system)	Wold et al. 2016 [7]
Bighead carp	Raman NMR	400–3500 cm^−1^ 22.6 MHz	PCA	Decrease in α-helix structures, reflecting changes in myosin secondary structures with increasing heat treatment. Water distribution and mobility of water change with thermal treatments	Yuan et al. 2018 [54]
False abalone	NMR, MRI	21.3 MHz	PLSR	Providing quantitative characterizations of actin and myosin protein denaturation and water distribution on the quality of the product	He et al. 2018 [49]
Turbot	NMR, MRI	21.2 MHz	PCA	Different water dynamics according to cooking method. Good correlation between NMR relaxation parameters and texture, color measurements. Internal structure visualized by MRI	Xia et al. 2017 [53]
White Shrimp	Raman	1600–1700 cm^−1^ 2200–3500 cm^−1^	Univariate analysis	Various bands of Raman spectra demonstrated changes in hydrogen bonding and protein denaturation	Gao et al. 2016 [68]
Hairtail surimi	FTIR, Raman	400–3500 cm^−1^ 300–1800 cm^−1^	Univariate analysis	Changes in secondary structures of protein, reflected in a decrease in α-helix and an increase in *β*-sheet structures, indicating protein denaturation induced by irradiation and heat treatment	Lin et al. 2015 [40]
Alaska pollock surimi	FTIR	4000–400 cm^−1^	PCA	Increasing cooking temperature reduced the gel strength of the surimi as a result of changes in protein secondary structures	Zhang et al. 2018 [69]
Atlantic salmon	FTIR	4000–400 cm^−1^	PCA	Amid I region revealed increased denaturation and aggregation of proteins with increasing cooking temperature and cooking time	Ovissipour et al. 2017 [6]
Atlantic salmon	FTIR	4000–400 cm^−1^	PCA	Cooking combined with electrolyzed water decreased strongly bacterial growth *of Listeria monocytogenes.* Higher protein denaturation and unfolding with increasing cooking temperature	Ovissipour et al. 2018 [70]
Hairtail	Fluorescence	Ex; 347 nm Em; 415 nm	Univariate analysis	Increased fluorescence was obtained in cooked fish as compared to raw samples. More fluorescence was noticed from baked and fried fillets as compared to boiled ones	Tavares et al. 2018 [71]
Sturgeon	Fluorescence	Ex; 360 nm Em; 380–600 nm	Univariate analysis	Roasting and frying cooking methods induced significant increases in fluorescence, as a result of formation of Schiff bases compounds, compared to boiling and steaming cooking methods	Hu et al. 2017 [44]
Sturgeon	Fluorescence	Ex; 360 nm Em; 380–600 nm	Univariate analysis	Fluorescence was increased after digestion (especially after gastrointestinal digestion) as compared to fluorescence of samples before digestion. Changes in spectral patters (shape and intensity) were observed with different roasting times	Hu et al. 2018 [11]
Atlantic mackerel	Fluorescence	Ex; 365 nm Em; 445 nm	Univariate analysis	Direct relationship between total collagen content and hardness, and total fluorescence intensity of collagenous tissue	Cropotova et al. 2018 [17]
Atlantic mackerel	Fluorescence	Ex; 475 nm Em; 530 nm	Univariate analysis	High correlation between fluorescence and lipid oxidation products (primary and secondary indicators of lipid oxidation)	Cropotova et al. 2019 [20]

VIS/NIR, Visible/Near; FTIR, Fourier-transform infrared spectroscopy; NMR, Nuclear magnetic resonance; MRI, Magnetic resonance imaging; PLSR, partial least squares regression; MLR, Multiple linear regression; LAD, Linear discriminant analysis; PCA, Principal component analysis; Ex, Excitation; Em, Emission.
